# Laccases: Versatile Biocatalysts for the Synthesis of Heterocyclic Cores

**DOI:** 10.3390/molecules26123719

**Published:** 2021-06-18

**Authors:** Ana Catarina Sousa, Lígia O. Martins, M. Paula Robalo

**Affiliations:** 1 Área Departamental de Engenharia Química, Instituto Superior de Engenharia de Lisboa, Instituto Politécnico de Lisboa, R. Conselheiro Emídio Navarro 1, 1959-007 Lisboa, Portugal; acsousa@deq.isel.ipl.pt; 2 Centro de Química Estrutural, Complexo I, Instituto Superior Técnico, Universidade de Lisboa, Av. Rovisco Pais, 1049-001 Lisboa, Portugal; 3 Instituto de Tecnologia Química e Biológica António Xavier, Universidade Nova de Lisboa, Av. da República, 2780-157 Oeiras, Portugal

**Keywords:** biocatalysis, heterocycles, oxidoreductases, bioprocesses, cross-coupling reactions, green methods, sustainability

## Abstract

Laccases are multicopper oxidases that have shown a great potential in various biotechnological and green chemistry processes mainly due to their high relative non-specific oxidation of phenols, arylamines and some inorganic metals, and their high redox potentials that can span from 500 to 800 mV vs. SHE. Other advantages of laccases include the use of readily available oxygen as a second substrate, the formation of water as a side-product and no requirement for cofactors. Importantly, addition of low-molecular-weight redox mediators that act as electron shuttles, promoting the oxidation of complex bulky substrates and/or of higher redox potential than the enzymes themselves, can further expand their substrate scope, in the so-called laccase-mediated systems (LMS). Laccase bioprocesses can be designed for efficiency at both acidic and basic conditions since it is known that fungal and bacterial laccases exhibit distinct optimal pH values for the similar phenolic and aromatic amines. This review covers studies on the synthesis of five- and six-membered ring heterocyclic cores, such as benzimidazoles, benzofurans, benzothiazoles, quinazoline and quinazolinone, phenazine, phenoxazine, phenoxazinone and phenothiazine derivatives. The enzymes used and the reaction protocols are briefly outlined, and the mechanistic pathways described.

## 1. Introduction

Heterocyclic compounds are important molecules among the applied branches of organic chemistry. They are abundant in natural products and their properties are useful in the design of several pharmaceuticals and new materials. They are key structural components in many molecular drugs, due to their ability to hydrogen bond and other properties, exhibiting an overall inhibitor effect retarding the progression of several diseases [1,2,3]. In the area of new materials, heterocycles can impart unique and useful electronic and optical properties [4,5,6]. A large number of N-based or O-based heterocycles have found additional utility as dyestuffs, copolymers, and valuable intermediates in synthesis. They display many advantages, including an easy preparation, low toxicity, low adverse effects, high bioavailability, low drug resistance and good biocompatibility. Therefore, the synthesis of heterocyclic compounds has attracted considerable interest in the last decades and a variety of synthetic protocols have been developed [7]. Despite the wide availability of synthetic methods, the development of new and more efficient procedures or methods is still required. Organic synthesis of chemicals suffers from several drawbacks, including the high cost of chemicals, cumbersome multi-step reactions and toxicity of reagents. Most reported methods in the literature involve the use of excess amounts of expensive and toxic oxidants at abrasive reaction conditions (high temperatures and pressure, as well as long reaction times) and environmentally unfriendly solvents [8,9,10].

The increased societal interest in products from renewable feedstocks, greener processes and the recent advances in biotechnology have brought the application of enzymes to the forefront of research to address the current challenges of modern synthetic organic chemistry. Enzymatic processes are green and sustainable, since biocatalysts are biocompatible, biodegradable and essentially non-hazardous and non-toxic. Enzymatic reactions generally avoid the need of conventional organic synthetic procedures such as functional group activation, protection and deprotection steps, affording routes with a lower number of steps, which are more cost-effective and generate reduced amounts of waste. Furthermore, enzymatic processes are in general quite selective, distinguishing between regio- and stereoisomers and discriminating various functional groups.

Enzymes have been continually expanding their catalytic applications in industrial, medical and diagnosis fields, owing to their high catalytic efficiency, substrate specificity, mild reaction conditions and good environmental safety [11,12,13]. Eco-friendly oxidation bioprocesses represent an attractive and important alternative to the traditional chemical synthetic methods in the green chemistry field, allowing the development of sustainable processes and production of new molecules.

Laccases couple the oxidation of a wide range of aromatic substrates with the reduction of molecular oxygen to water. They are very interesting biocatalysts that have attracted considerable attention in the last decades in environmental and biotechnological processes, including drug, food, textile, cosmetics, and biodegradation of organic compounds in wastewater, enzymatic biofuel cells, among others [14,15,16,17,18,19]. Laccase reactions, which promote aromatic compounds oxidation in the presence of oxygen as a co-substrate, do not use toxic reagents and do not display hazardous side effects, have received increasing attention in the synthesis of fine chemicals. The type of chemical transformations that can be performed and the chemical structures that can be accessed are vast and can be further broadened by laccase-mediator systems (LMS) [13,20,21,22,23,24]. 

In the present review, the contribution of the laccase-assisted biocatalytic processes as alternative approaches to the synthesis of N-, S- and O-based aromatic heterocycles will be described. The review is organized according to the main heterocycle types in order of increasing complexity, ring size, number of heteroatoms and their fused analogues.

## 2. Biocatalysis with Laccases

### 2.1. Laccases Are Widespread Enzymes

The laccase from the lacquer tree *Rhus vernicifera* was the first laccase described and is responsible for the oxidation of urushiol, a milky secretion of the lacquer tree, in the presence of air by a process of polymerization and cross-linking producing lacquer, a hard and strong resin that has been widely used in traditional oriental crafts [25]. Plant laccases are found in the xylem where they oxidize monolignols in the early stages of lignification [26] and contribute to the cross-linking of cell wall structural proteins [27]. The vast majority of laccases characterized so far have been, however, isolated from fungi, in particular white-rot basidiomycetes, where they play a role in lignin degradation [28]. Fungal laccases can also act as a virulent factor such as the grapevine grey mould and the chestnut blight fungus [26] and have been described as prominent virulence factors in pathogenic yeast [29]. In the eukaryotic domain, laccases are also present in insects, where they are active in the cuticle sclerotization [28]. In the last two decades, a large number of laccases of bacterial origin have been identified and characterized [30,31,32,33]. Their role has been assigned to the microorganisms’ copper resistance, morphogenesis, sporulation, pigmentation, lignocellulose degradation, bacteria–bacteria interactions or antibiotic production [34].

### 2.2. Overall Structure of Laccases and Catalytic Mechanisms

**Figure 1:**
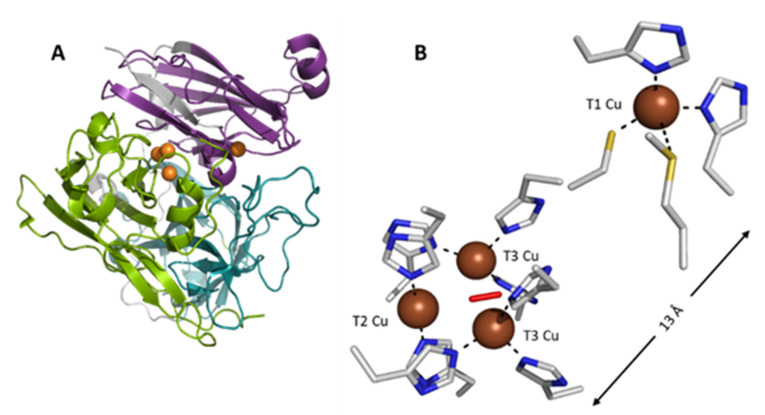
(**A**) Representation of the three-dimensional structure of CotA laccase with the cupredoxin domains coloured differently (residues 1–173, domain1: green; residues 182–340, domain2: blue; residues 369–501, domain 3: violet). The four copper atoms are shown as orange spheres. (**B**) The mononuclear T1 centre is on the right and the trinuclear centre is on the left. Pictures drawn with the use of PyMOL software and supported by the deposited structure in Protein Database PDB1w6l.

Laccases belong to the family of multicopper oxidases (MCOs) that typically have an overall structural fold comprising three cupredoxin-type domains with a Greek key β-barrel topology (Figure 1A) [35]. MCOs contain four Cu atoms, the T1 Cu site involved in substrate oxidation, and T2 and T3 Cu atoms that form a trinuclear centre (TNC); they couple the one-electron oxidation of substrates at the T1 Cu with the four-electron reduction of molecular oxygen to water at TNC (Figure 1B) [36,37,38]. The T1 Cu is coordinated by two histidine nitrogen atoms and a cysteine sulphur, and it is characterised by an intense S(π)→Cu(dx^2^−y^2^) charge transfer absorption band at around 600 nm, ε_600 nm_ > 3000 M^−1^ cm^−1^ responsible for the intense blue colour of the enzymes. The T2 copper site, strategically positioned close to the T3 binuclear copper centre, is usually coordinated by two histidine residues and a water (or hydroxyl) molecule, while each T3 copper is coordinated by three histidines and a bridging ligand such as a hydroxyl moiety, displaying an absorption in the near-UV, with λ_max_ = 330 nm. The mononuclear T1 Cu site interacts with the trinuclear cluster T2/T3 through the highly conserved HCH motif, where the cysteine in the T1 binding Cu shuttles electrons over a distance of ∼13 Å to each of the two histidines coordinated to T3 copper ions (Figure 1B). The reaction mechanism of laccases and other MCOs have been extensively studied by biochemical, kinetic, spectroscopic, and structural techniques [39]. The main electron transfer steps in the reaction mechanism are the (i) reduction of the T1 Cu site by the oxidized substrate, (ii) electron transfer from the T1 Cu site to the trinuclear cluster, and (iii) O_2_ reduction by the trinuclear cluster. The T1 Cu centre is sited at the bottom of the substrate binding region, relatively exposed to the solvent, and interacts with substrates through the imidazole ring of one of its His ligands [40,41,42,43]. The broad range of organic substrates capable of being oxidized by MCOs is a result of non-covalent binding near the T1 Cu for outer-sphere electron transfer (ET) [39].

### 2.3. Bacterial Versus Fungal Laccases: Redox Potential and pH Optima

Laccases have nearly identical Cu active sites, but they exhibit significant differences in substrate specificity and catalytic rates. These differences have been assigned to alterations in second-sphere residues around the T1 Cu centre. The vast majority of studies and applications were performed using fungal laccases that show, in general, higher redox potentials (around 800 mV vs. SHE). Bacterial laccases (with E^0^ around 500 mV vs. SHE) also show interesting properties for diverse biotechnological applications, such as higher thermostability and optimal pH values in the neutral to basic range, in contrast with fungal laccases that operate maximally in the acidic range of pH.

A high redox potential increases the range of oxidizable substrates and improves the effectiveness and versatility of the enzyme. In laccases, such as those of bacterial origin, which bear a T1 Cu methionine axial ligand, the copper lies above the plane defined by the nitrogen and cysteine sulphur ligands and is displaced towards the methionine, showing a distorted tetragonal geometry [37]. Fungal laccases have non-coordinating phenylalanine or leucine at this position, favouring a trigonal planar geometry for the site, which is believed to contribute to the higher redox potential observed in these enzymes [44,45,46]. The replacement of the axial ligand (residue Met502) at the T1 site of CotA by leucine and phenylalanine led to an increase in the E^0^ by c.a. 100 mV, although the higher E^0^ determined did not favour an increased oxidation rate, since the mutations had a profound impact on the stability of the enzyme [44]. Conversely, mutation of the axial ligand (residue F463) to methionine in the *Trametes villosa* laccase decreases the redox potential from 790 to 680 mV [46]. Structural studies on the *Trametes trogii* laccase (E^0^T1 = 760 mV) suggested an important contribution for the hydrophobic residues near the T1 copper site to the high redox potential observed for this enzyme [43]. Similar conclusions have been reached by the experimental replacement of I494 and L386 hydrophobic residues in the vicinity of the T1 copper site of the CotA laccase by alanines that led to a lower E^0^ due to an increase in the solvent accessibility to this centre, stabilizing the T1 copper in the +2 oxidation state [47]. Overall, the available literature indicates that the variations in redox potential of the T1 centre observed among laccases is not assigned to a single structural feature but to a sum of factors such as the copper centre coordination geometry and the nature of the second sphere residues influencing solvent accessibility, hydrogen bonding, and dielectric anisotropy around the site.

**Figure 2:**
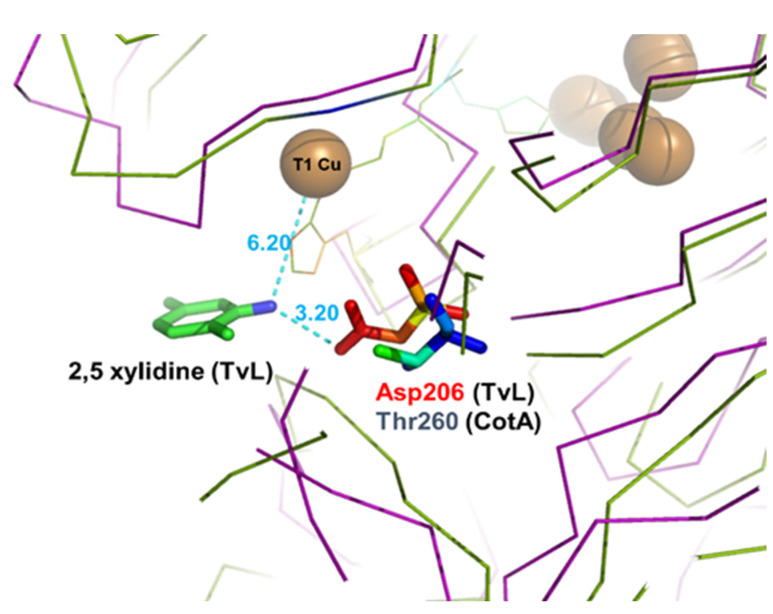
Overlap of the active site of the fungal TvL and bacterial CotA laccases with detail on the Asp206/Thr260. Pictures drawn with the use of PyMOL software and supported PDB1w6l and 1KYA. It is believed that carboxylate residues are crucial to oxidise substrates containing phenolic or aromatic amine functional groups aiding in the deprotonation of substrates.

Laccases exhibited different optimal pH values for different substrates. For substrates, which involves the release of a proton and an electron (such as phenolics and arylamines), laccases have a bell-shaped pH activity profile with an optimal pH dependent on the laccase and the substrate [48]. This is consistent with a mechanism that balances two opposing effects, one generated by the redox potential difference between the reducing substrate and the T1 Cu (correlating to the electron transfer rate, favoured by higher pH), and another generated by the binding of a hydroxide anion to the T2/T3 Cu (which inhibits the activity at a higher pH) [48]. Interestingly, fungal laccases such as the *Trametes versicolor* (TvL) laccase show maximal rates at the acidic range, while bacterial laccases show a clear preference for the basic range of pH values [49]. All well-characterized fungal laccases have a conserved Asp or Glu residue close to the substrate binding site cavity that is not present in CotA or in any bacterial laccase identified so far (Figure 2). The negative charge close to the active site in TvL (Asp 206) and in *Melanocarpus albomyces* (Glu 235) was proposed to have a role in facilitating substrate oxidation by accepting a proton from the substrate [40,50,51]. In the case of laccases that do not contain any negatively charged residue in the vicinity of the substrate binding site (Figure 2), such as CotA, the efficiency of the oxidation relies mostly on the protonation/deprotonation state equilibria of the compounds themselves [49,52,53]. Furthermore, maximal rates of oxidation are dependent on the electronic nature of other substituents, which are key factors for the stability of the radicals formed; the presence of electron-withdrawing substituents leads to a higher stabilization of radicals which, as expected, impacts positively on the rates of enzymatic oxidation [49].

### 2.4. Laccases-Mediated Reactions

**Scheme 1:**
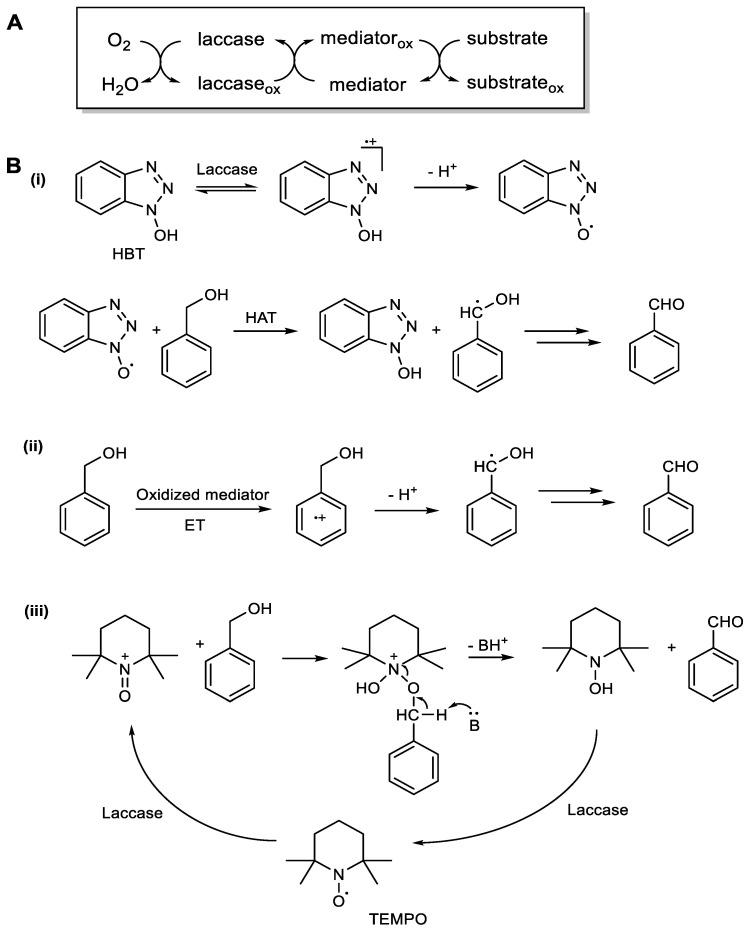
Schematic representation of (**A**) the laccase-mediator redox cycle; (**B**) of the mediator–substrate oxidation via the (i) HAT route, (ii) the electron transfer (ET) route and (iii) the ionic oxidation route. Adapted from [20].

The substrate scope of laccases can be enhanced in the presence of small redox mediator molecules in the so-called laccases-mediated systems (LMS). In reactions where the substrate has a higher E^0^ than the laccase or is too large to penetrate into the enzyme active site, the presence of redox mediators may facilitate reactions [54]. The mediator should be a substrate of the enzyme that, upon reaction, forms a reactive oxidized intermediate which, then, diffuses away from the enzymatic pocket and oxidizes the substrate by mechanisms different from the enzymatic one (Scheme 1A). Ideally, a redox mediator should generate stable radicals in its oxidized form that do not inactivate the enzyme, and whose reactivity would allow its recycling without degradation. The mechanism of the mediator–substrate oxidation varies with the redox mediator molecule used [20,22,55]. Mediators that have the N–OH structural feature, such as HBT (1-hydroxybenzotriazole), favour the radical hydrogen-atom transfer (HAT) pathway, while ABTS (2,2′-Azino-bis(3-ethylbenzothiazoline-6-sulfonic acid) diammonium salt) reacts via an electron transfer (ET) route. Other mediators, such as TEMPO (2,2′,6,6′tetramethyl piperidine N-oxyl) and its analogues, are suggested to follow an ionic oxidation pathway (Scheme 1B). Despite the proven efficiency of LMS systems to assist laccases’ reactions, the application of these systems is partially hindered by their cost and the generation of possible toxic species. This led to an interest in understanding which mediator’s laccase uses in nature since it is thought that the biodegradation of the non-phenolic aromatic structures of lignin by fungal laccases occurs by a process that involves free radicals, derived from their own biodegradation process, acting as redox mediators. The description of the fungal metabolite 3-hydroxyanthranilic (3-HAA) as a mediator was one of the first evidences of the contribution of redox mediators of natural origin to assist lignin biodegradation [56]. The enzymatic oxidation of several polycyclic aromatic hydrocarbons (PAHs) mediated by other fungal phenolic metabolites was also achieved with, i.e., 4-hydroxybenzoic acid and 4-hydroxybenzylic alcohol [57]. The mediator 4-hydroxybenzoic acid has been also used for fungicide degradation and detoxification [58] as well as syringaldehyde, acetosyringone, vanillin, among other naturally occurring substituted phenols related to lignin, [59] although the stability of the corresponding phenoxy radicals does not favour their wide utilization.

## 3. Application of Laccases in Bio-Oxidative Synthesis of Heterocyclic Compounds 

The vast majority of laccase biocatalytic synthetic processes have been reported using phenols as substrates. The reactions involve the radical-coupling of phenolic monomers and cross-coupling of substituted catechols and hydroquinones with nitrogen-based nucleophiles via in situ generated *ortho*- and *para*-quinones and have provided new synthetic routes to aminoquinones and other C–N coupling derivatives [9,60,61,62,63,64]. Far less reported are the oxidative reactions of laccases with a wide range of different aromatic amines and their radical-coupling reactions, involving the generated *o*- and *p*-benzoquinonediimine or benzoquinoneimine intermediates, leading to relevant bio-products. Noteworthy, the dual behaviour of these compounds as substrates vs. nucleophiles is a key feature for investigating alternative synthetic approaches to the synthesis of heterocyclic compounds [65,66,67].

**Figure 3:**
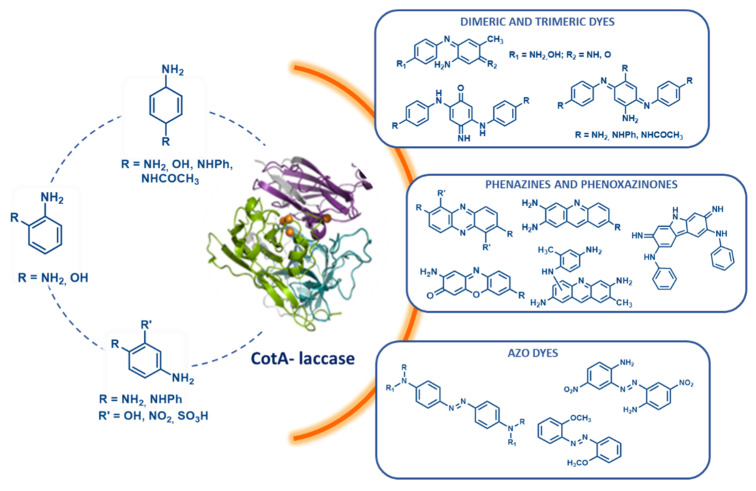
Aromatic scaffolds obtained from oxidation of structurally different aromatic amines using CotA-laccase.

Several laccases have been employed in the enzymatic synthesis of oligomers of arylamines, those of fungal origin such as from *T. versicolor*, *Pycnoporous cinnabarinus*, *Pleurotus ostreatus*, *Cerrena unicolor*, *T. villosa*, *Myceliophthora thermophila*, *Agaricus bisporus*, as well as the bacterial CotA laccase from *Bacillus subtilus* (see Table 1). The main products and the product distribution were shown to be critically dependent of the reaction conditions employed, namely ratio of laccase/arylamine concentration, buffer medium, the presence of organic co-solvents, the pH and temperature conditions and duration of reactions. We have undertaken systematic studies using a wide range of substituted arylamines and the bacterial CotA-laccase [65,66,67,68,69,70] that showed the importance of the substitution pattern and the electronic nature of the substituents in the product distribution as well as the type of structures to be obtained (Figure 3). The efficiency of the CotA laccase enzymatic system was found to be strictly dependent on (i) the difference between the redox potential of the enzyme (550 mV) [71] and the substrates, and (ii) the pH of the reaction that affects both the catalytic activity of laccase and the redox potentials of the substrates, i.e., their susceptibility for oxidation [68]. For example, the susceptibility to enzymatic transformation relies on the electron density at the amino group and electron-donating substituents increasing the yields of reaction; likewise, anilines substituted by electron-accepting groups in *para* positions did not undergo enzymatic transformations [68]. The CotA-laccase oxidation of *o*-phenylenediamines, substituted *p*-diphenylamines and *o*-aminophenols, among others, at the neutral to the basic range of pH, yielded dimeric and trimeric dyes [68,69,70] as well as substituted heterocyclic frameworks (phenazine, phenoxazinone, carbazole derivatives) [65,66,67] at the neutral to basic range of pH values (Figure 3). The formation of azo dyes was also observed as secondary products of laccase´s biotransformation [65,67,68] or as the main products using appropriate arylamines as substrates in the presence of ABTS as mediator [70].

Regarding the cyclization reactions catalysed by laccases, several reports in the last decades have arisen on the formation of several nitrogen-based heterocyclic cores (benzimidazoles, benzothiazoles, quinazoline and quinazolinone derivatives, phenazines, phenoxazine and phenoxazinones, phenothiazines and benzothiadiazine-8-ones) as well as some oxygen based heterocyclic moieties (benzoxazoles and benzofurans). The most relevant synthetic pathways for N-based and O-based heterocyclic compounds mediated by laccases are listed in Table 1, which summarize also the optimized reaction conditions and obtained yields. Most of these heterocyclic aromatic compounds are important active pharmaceutical ingredients, associated to a wide range of biological and pharmacological activities such as anti-tumour, anti-fungal, antiviral, anti-allergic, antidepressant, antioxidant, anti-HIV, anticonvulsant, anti-diabetic, antipsychotic, anti-malarian and anti-inflammatory activities [10,22,72,73,74,75,76,77].

### 3.1. Synthesis of Five-Membered Ring Heterocycles

#### 3.1.1. Synthesis of Benzofuran-Based Heterocycles

**Scheme 2:**
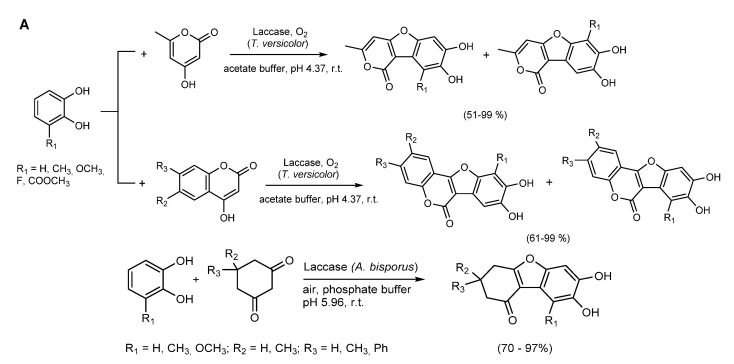

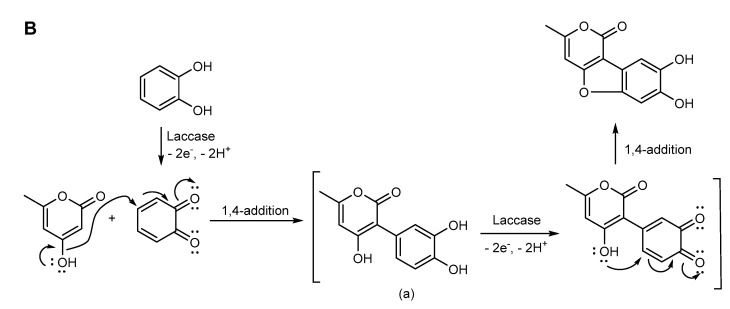
(**A**) Laccase initiated domino reactions with catechols and 1,3-dicarbonyl compounds [78,79]; (**B**) Mechanistic proposal for the reaction [78].

Benzofurans represent one of the most studied families of O-heterocycle compounds owing to their relevance as potential natural drug lead compounds. The oxidative capacity of laccases was explored to mediate the synthesis of benzofuran derivatives through cascade reactions between catechols and 1,3-dicarbonyl compounds. The formation of coumestans and related O-heterocycles was reported using the *T. versicolor* laccase as a biocatalyst for the domino reactions between 4-hydroxy-6-methyl-2H- pyran-2-one or substituted 4-hydroxy-2H-chromen-2-ones and catechols (Scheme 2A) [78]. A number of different heterocyclic systems were also reported with the *A. bisporus* laccase in domino reactions between catechols and several cyclic and heterocyclic (pyridinones, quinolinones, thiocoumarins) 1,3-dicarbonyls [79,80,99]. For example, the reactions of cyclohexane-1,3-diones with catechols in the presence of the *A. bisporus* laccase afforded 3,4-dihydro-7,8-dihydroxy-2H-dibenzofuran-1-ones with yields ranging from 70% to 97% (Scheme 2A) [79].

As outlined in Scheme 2B, the first step of the reaction is the laccase-catalysed oxidation of catechol to give the *o*-benzoquinone, which then reacts with the nucleophilic 4-hydroxy-6-methyl-2H-pyran-2-one in an intermolecular 1,4-addition leading to the non-isolable intermediate (a). After a second laccase-catalysed oxidation of (a), an intramolecular 1,4-addition occurs giving the final heterocycle. Altogether, a domino oxidation/1,4-addition/oxidation/1,4-addition process takes place [78,79].

**Scheme 3:**
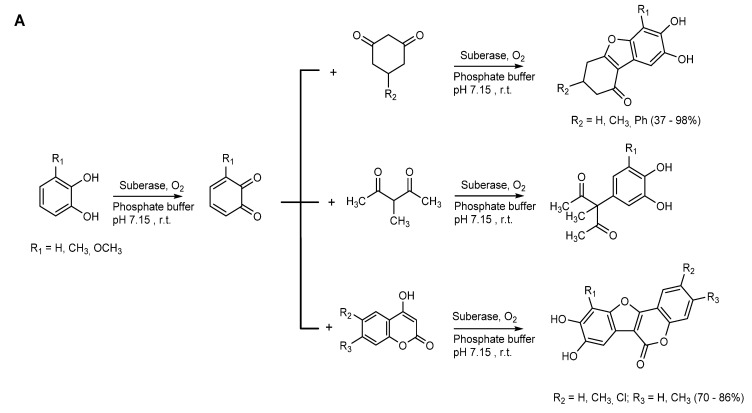

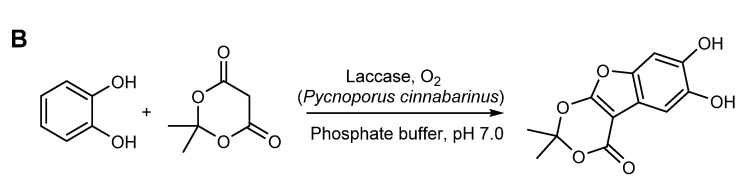
Synthesis of the C–O and C–C coupled products assisted by laccases. (**A**) Synthesis of the 5,6-dihydroxylated benzo[b]furans, C–C coupled products and coumestans [81,82]; (**B**) Synthesis of 6,7-dihydoxy-2,2-dimethyl-1,3,9-trioxa-fluorene-4-one using catechol and Meldrum’s acid [83].

Inspired by these initial studies, more work has been successfully performed with the *M. thermophila* laccase Suberase^®^ [81,82] and the *P. cinnabarinus* laccase [83] for the oxidation–Michael addition of catechols and aliphatic, cyclic, and heterocyclic 1,3-dicarbonyls for the synthesis of a variety of benzofuran derivatives (Scheme 3). Oxidations occurred in a non-stereoselective mode but with complete regio- and/or monoselectivity and products were obtained at excellent purity after a simple extraction. Overall, these studies exemplify the versatility of the laccase-initiated cascade reactions as an useful synthetic tool for organic chemists.

#### 3.1.2. Synthesis of 2-Arylbenzimidazoles

Benzimidazoles and structurally related compounds occupy a pivotal position in medicinal chemistry and the efficient synthesis of benzimidazoles and their derivatives remains highly important and a rewarding target for synthetic organic chemists [100,101].

**Scheme 4:**
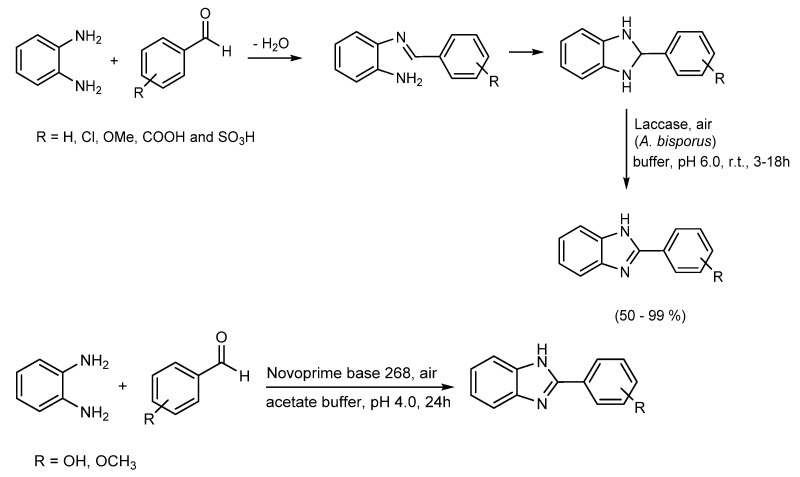
Laccase-catalysed reaction of *o*-phenylenediamines with aromatic aldehydes to yield 2-aryl-1H-benzimidazoles [10,72].

Greener approaches for the formation of benzimidazole derivatives have been reported, including a laccase-catalysed domino reaction between *o*-phenylenediamine (1,2-PDA) and substituted benzaldehydes that exclusively afforded 2-aryl-1H-benzimidazoles in good to very good yields (Scheme 4) [72]. The reaction was suggested to start with the formation of the Schiff base from the reaction of 1,2-PDA with aldehyde, followed by an intramolecular ring closure to produce the N,N-acetal. In the second step, the laccase-catalysed oxidation of the acetal yielded the benzimidazole. The formation of the 1H-benzimidazole ring system was selective under the reaction conditions used, since no dimerization of *o*-phenylenediamine into the 2,3-diaminophenazine was observed [72].

The one-pot synthesis of 2-aryl-1H-benzimidazoles in good to excellent yields (56–88%) was reported using the commercial laccases Novoprime Base 268, Suberase^®^ and Denilite^®^ II Base at room temperature [10]. The selectivity of the reactions of *o*-phenylenediamine with aryl aldehydes, bearing both electron-donating and electron-withdrawing substituents, was studied, by varying several reaction conditions and the use of acetonitrile as co-solvent was found to promote the selective formation of the 2-aryl-substituted benzimidazoles (Scheme 4) [10].

**Scheme 5:**
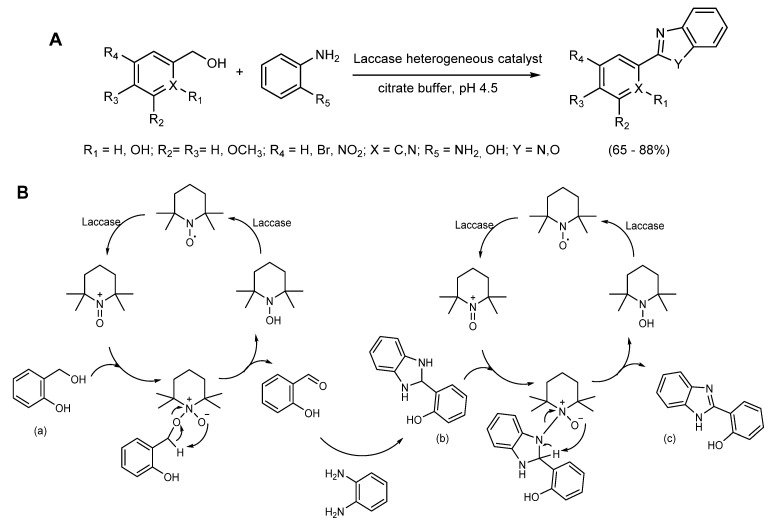
(**A**) Synthesis of benzimidazoles by heterogeneous laccase-catalysed aerobic oxidation of salicyl alcohol; (**B**) Plausible mechanism for laccase/TEMPO catalysed aerobic oxidation of salicyl alcohol (a) followed by an oxidative cyclization reaction and further biocatalytic aerobic dehydrogenation of intermediate (b) to produce 2-hydroxybenzimidazole (c).

An elegant enzymatic oxidative cascade reaction was designed to synthesise benzimidazole (or benzoxazole) derivatives from salicyl alcohol using the *T. versicolor* laccase and the mediator TEMPO immobilized separately on amine functionalized iron(II,III) oxide nanoparticles [84]. Enzyme immobilization on magnetic nanoparticles allows an easy, fast and clean separation of products, increasing the efficiency of catalytic LMS. In the first step, aldehydes with electron-withdrawing groups were obtained in relatively higher yields when compared to aldehydes bearing electron-donating groups. This process was followed by the condensation of *in situ*-produced salicylaldehyde derivatives with *o*-phenylenediamine (or *o*-aminophenol) followed by a biocatalytic aerobic dehydrogenation process under mild reaction conditions to synthetise benzimidazole (or benzoxazole) derivatives (Scheme 5A) [84].

The proposed mechanism involves the disproportionation of TEMPO oxidized by the laccase to form an oxoammonium ion at acidic conditions (Scheme 5B). The oxidized TEMPO oxidizes the alcohol (a) via simultaneous reduction to hydroxylamine to produce the corresponding aldehyde. This intermediate suffers a nucleophilic addition by the arylamine to generate the intermediate (b) and produce the final 2-hydroxybenzimidazole.

The reaction conditions were optimized (pH, temperature, incubation time, concentration of reactants and organic solvents) and the recyclability of the catalytic LMS showed up to 85% retention of initial activity after 10 runs. In addition to the potential for reuse without significant losses in performance, other eco-friendly attributes of this catalytic LMS include its high conversion yields and its ease recovery from the reaction mixtures using magnets.

#### 3.1.3. Synthesis of Benzothiazoles

Benzothiazoles are members of the family of fused heterocycles that have attracted much attention due to their medical applications. The most popular approach for the synthesis of benzothiazoles is the condensation of 2-aminothiophenols with aldehydes under oxidative conditions.

**Scheme 6:**
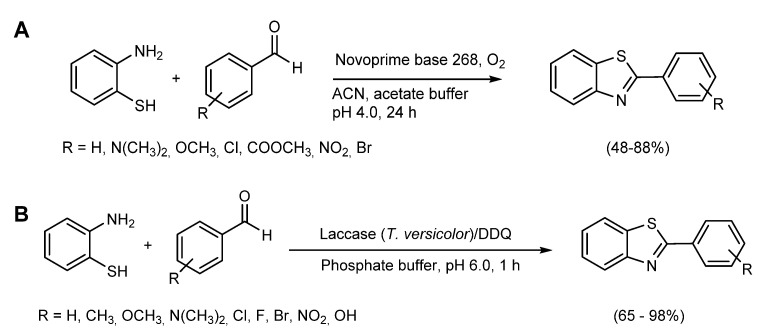
Reaction between 2-aminothiophenol and aldehydes to afford 2-arylbenzothiazoles using (**A**) Novoprime base 268 laccase [10] and (**B**) a laccase (*T. versicolor*)/DDQ catalytic system [85].

The laccase-catalysed cross-coupling reaction between the 2-aminothiophenol with several substituted aldehydes to afford 2-phenylbenzothiazoles at pH 4.0 and in the presence of 50% of acetonitrile as a cosolvent was reported (Scheme 6A) [10]. More recently, the *T. versicolor* laccase was used in a cooperative catalytic system with 2,3-dichloro-5,6-dicyano-1,4-benzoquinone (DDQ) for the synthesis of 2-arylbenzothiazoles (65–98% yield) via oxidative cyclization of Schiff bases derived from the condensation of 2-aminothiophenol with aldehydes (Scheme 6B) [85]. Numerous aldehydes such as benzaldehydes bearing electron-donating and electron-withdrawing groups, heterocyclic and α-β,unsaturated aldehydes, naphthaldehydes and 9-anthraldehyde were successfully applied to prepare the corresponding products via the reaction with 2-aminothiophenol, although in some cases the reactions were incomplete.

**Scheme 7:**
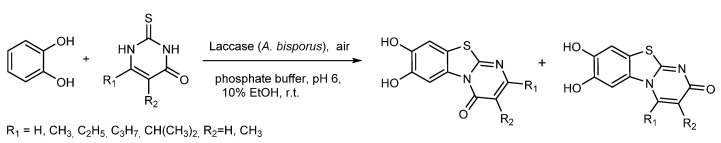
Laccase-catalysed reaction between catechol and 2,3-dihydro-2-thioxopyrimidin -4(1H)-ones [86].

Looking forward the synthesis of novel pyrimidobenzothiazoles with potential anticancer activity, a laccase-catalysed method was set-up using a commercial laccase from *A. bisporus* [86]. Catechol and 2,3-dihydro-2-thioxopyrimidin-4(1H)-ones were used as substrates to synthesize pyrimidobenzothiazoles (one of the possible regioisomers) but, although the high yields of the regioisomeric mixtures (up to 97%), generally the reactions were not selective (Scheme 7) [86].

### 3.2. Synthesis of Six-Membered Ring Heterocycles

#### 3.2.1. Synthesis of Quinazoline and Quinazolinone Derivatives

**Scheme 8:**
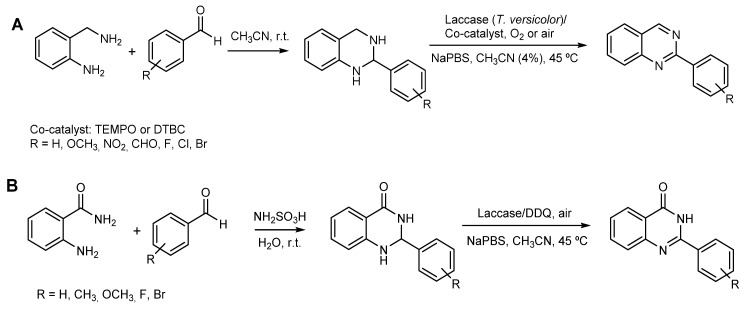
(**A**) Aerobic oxidative synthesis of 2-arylquinazoline and (**B**) quinazolin-4(3H)-ones catalysed by laccase (*T. versicolor*)/co-catalyst LMS [85,87].

Quinazoline and quinazolin-4(3H)-ones are important nitrogen-containing heterocycles and the most convenient method for the synthesis of these valuable compounds is the cyclization of *o*-anthranilamides with aldehydes followed by subsequent oxidation [85,87]. Saadati et al. reported a simple and efficient method for the synthesis of 2-substituted quinazolines through a cascade reaction of 2-aminobenzylamine and structurally diverse aldehydes via aerobic oxidative cyclization at pH 4.5 in the presence of laccase/3,5-di-tert-butylcatechol (DTBC) and laccase/TEMPO catalytic LMS (Scheme 8A). The oxidative system showed to be compatible with the presence of various substituents at different positions of the benzaldehyde ring and gave the desired products in moderate to high yields (40–96%). The same catalytic systems showed to be effective for the synthesis of other heteroaromatics such as quinoxaline, quinoline, indole and Hantzsch-type pyridine from aerobic dehydrogenation of their partial saturated precursors [87].

Recently, a laccase (from *T. versicolor*)/DDQ bioinspired cooperative catalytic LMS was used for the synthesis of quinazolin-4(3H)-ones (80–95% yield) in aqueous media at ambient temperature [85]. The chemoenzymatic synthesis of quinazolinones occurs in a two-step sequence: (i) chemical cyclization of *o*-anthranilamide with aldehyde in the presence of sulfamic acid to afford 2,3-dihydroquinazolin-4(1H)-one, and (ii) chemoenzymatic aerobic oxidation in the presence of laccase/DDQ catalyst system (Scheme 8B).

The scope of the process was examined by replacing substituted benzaldehydes and the results showed that both aromatic aldehydes containing electron-donating (methyl and methoxy) and electron-withdrawing (fluoro and bromo) groups were efficiently converted to the respective products in very good to excellent yields (80–95%).

#### 3.2.2. Synthesis of Phenazine Derivatives

Phenazine cores are multifunctional and versatile building blocks widely distributed in a vast array of biologically active compounds. Due to their importance and broad field of applications, the development of new greener (bio)synthetic methodologies is crucial as an alternative to chemical routes for the formation of these aromatic frameworks.

**Scheme 9:**
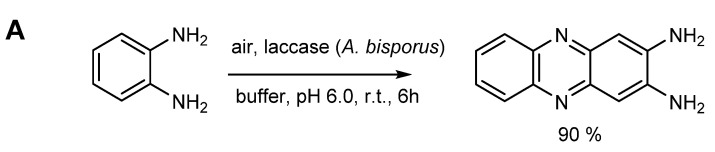

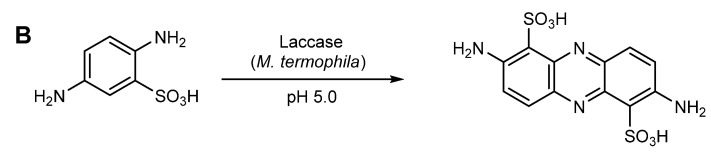
(**A**) Synthesis of 2,3-diaminophenazine and (**B**) 2,7-diaminophenazine-1,6-disulfonic acid by laccase-catalysed aerobic dimerization [72,88].

The oxidative transformation of *o*-phenylenediamine, under very mild reaction conditions, in the presence of catalytic amounts of a commercial laccase from *A. bisporus* exclusively delivered 2,3-diaminophenazine in 90% yield (Scheme 9A) [72]. The oxidative dimerization of 2,5-diamino-benzenesulfonic acid by the *M. termophila* laccase resulted in the formation of the 2,7-diaminophenazine-1,6-disulfonic acid (Scheme 9B) [88].

**Scheme 10:**
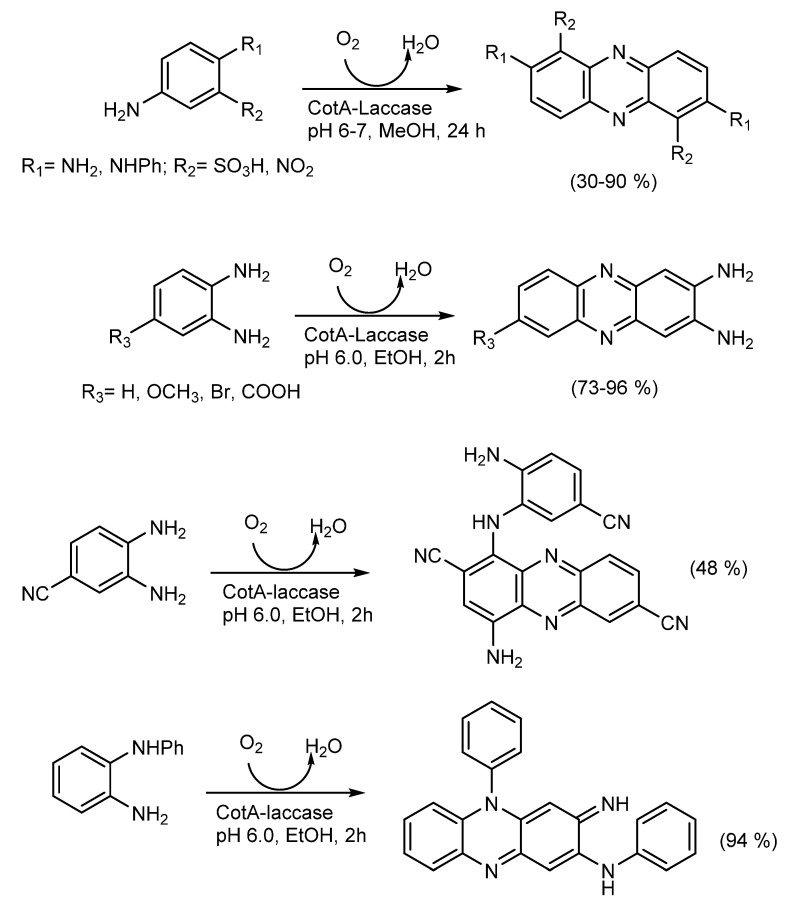
Synthesis of phenazine derivatives by oxidative homocoupling reactions of aromatic amines mediated by CotA-laccase [65,66,67].

The formation of different heterocyclic scaffolds, e.g., symmetric and asymmetric phenazines, phenoxazinones and carbazoles by oxidation of structurally different aromatic substrates assisted by the bacterial *B. subtilis* CotA-laccase was also reported by us [65,66,67]. The CotA-laccase oxidative homocoupling reactions of *ortho-para*- or *meta-para*-disubstituted aromatic amines resulted in different symmetric and asymmetric phenazines (Scheme 10) with good to excellent overall conversion yields.

**Scheme 11:**
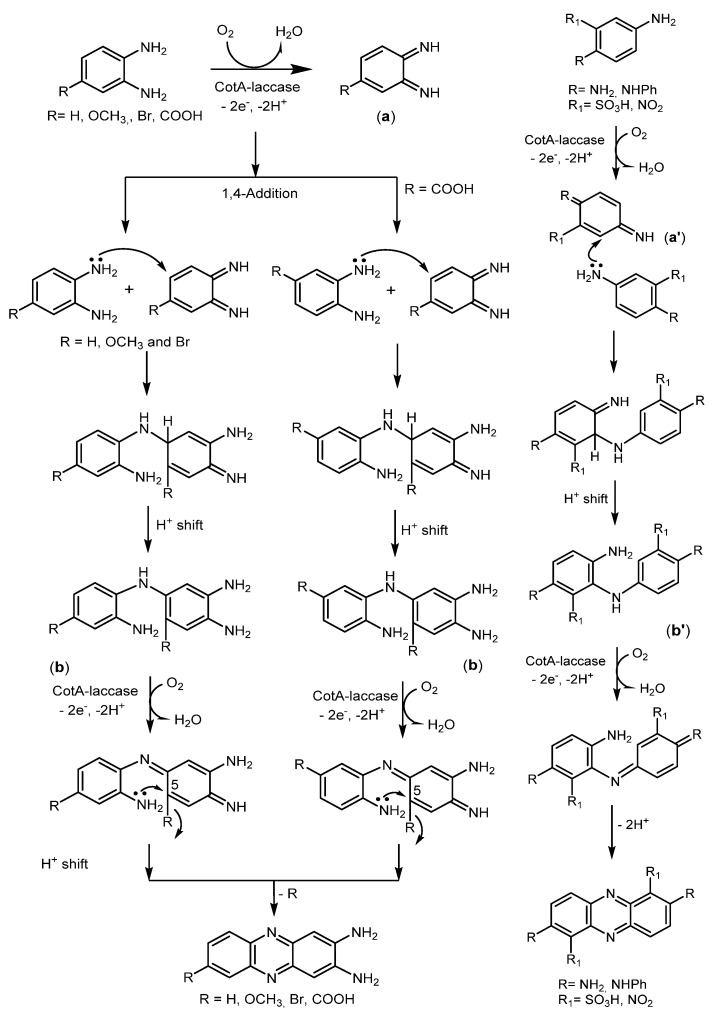
Proposed pathways involved in the formation of symmetric and asymmetric phenazines from substituted aromatic amines by CotA-laccase (adapted from [65,67]).

A mechanistic pathway was proposed (Scheme 11) where the initial step of the enzymatic process is the two successive one-electron oxidations of the *ortho*-diamines generating the *ortho*-quinone-diimine intermediates (a). These species suffer rapid nucleophilic addition by other substrate molecules in its most electrophilic carbon atom, followed by a proton shift, yielding the first coupling intermediate (b). The second two-step one electron oxidation is enzymatic and an intramolecular Michael addition of an amino group to the C5 atom, with the displacement of an R group, leading to aminophenazines, which are spontaneously oxidised in air to produce the final asymmetric heterocyclic products [65,94]. For the *meta-para*-disubstituted aromatic amines, the first step is the in situ generation of a *para*-benzoquinonediimine intermediate (a’) in a similar way as described above. This intermediate further reacts with the nucleophilic amino group of another molecule at the *ortho* position, adjacent to the R_1_ group resulting in the formation of dimeric structures. This second step, followed by a proton loss, yields the first coupling intermediate (b’). This non-isolable product underwent a subsequent oxidation, probably mediated by laccase, followed by an intramolecular Michael addition to form the symmetric substituted phenazines.

**Scheme 12:**
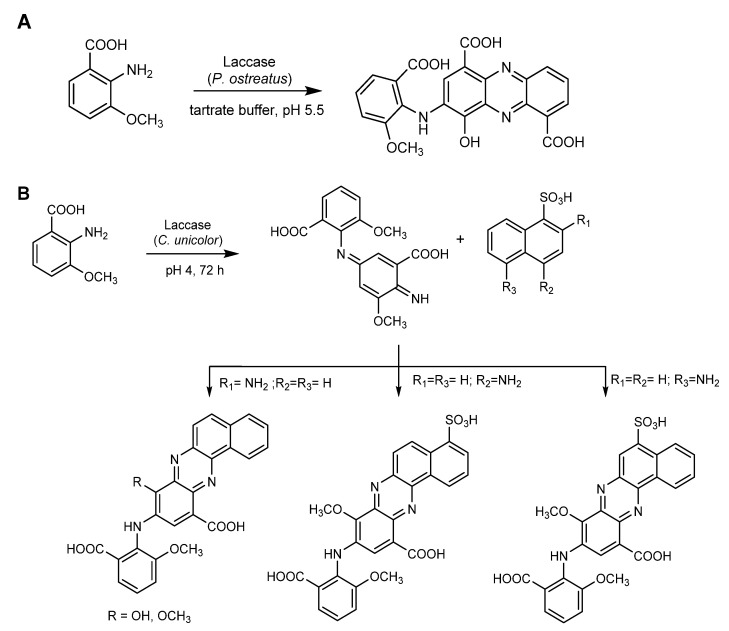
Synthesis of phenazines as a result of a laccase-mediated (**A**) homocoupling reaction and (**B**) heterocoupling reactions of 2-amino-3-methoxybenzoic acid [89,90].

The formation of a phenazine based orange dye by the homomolecular transformation of the 2-amino-3-methoxybenzoic acid in the presence of free and immobilised laccase from the *P. ostreatus* strain was very recently reported (Scheme 12A) [89]. Interestingly, the enzyme, when immobilised on Purolite^®^ carriers, showed a remarkable storage stability (21 days) and thermostability at 40 °C and 60 °C as compared to its free form. The same substrate 2-amino-3-methoxybenzoic acid can be involved in heterocoupling reactions with aminonaphthalene sulfonic acid isomers, leading to phenazine dyes, with the *C. unicolor* laccase, in mild conditions of pH, temperature and pressure (Scheme 12B) [90]. These dyes exhibited excellent dyeing properties as well as antibacterial and antioxidative activities; therefore, the proposed enzyme-mediated synthesis represents an alternative eco-friendly route for the synthesis of novel antimicrobial compounds with high importance for the medical textile industry.

#### 3.2.3. Synthesis of Phenoxazine and Phenoxazinone Derivatives

Phenoxazines and phenoxazinones are important classes of heterocyclic compounds containing a tricyclic iminoquinone core structure, also being an important building block present in compounds displaying significant biological activities and redox properties. Simple 2-aminophenoxazin-3-ones and 3-aminophenoxazin-2-one exhibit antitumor, antimicrobial, and antiviral activity in vitro and in vivo [75,102,103,104,105]. Due to their importance, a variety of synthetic procedures has been described [106,107].

**Scheme 13:**
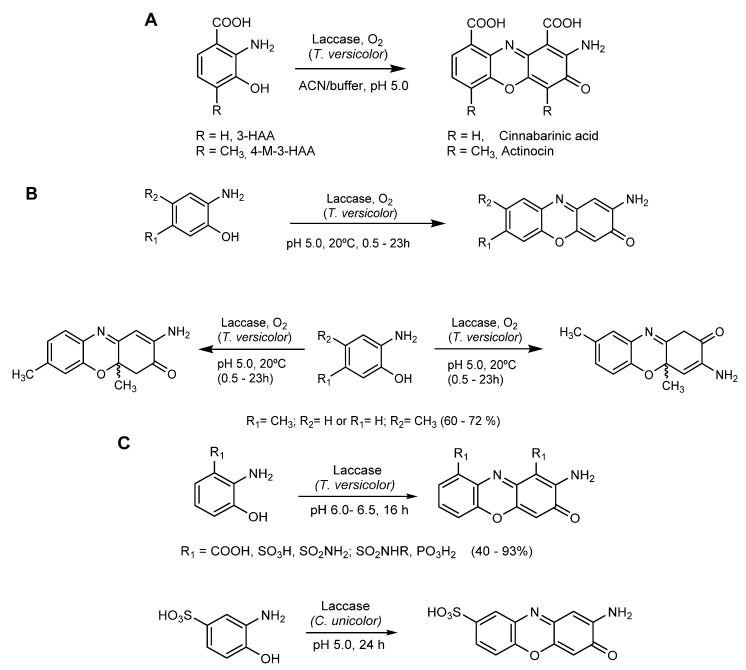
(**A**) Oxidation of 3-hydroxyanthranilic acid (3-HAA) and 4-methyl-3-hydroxyanthranilic acid (4-M-3-HAA) to cinnabarinic acid and actinocin, respectively, using *T. versicolor* laccase [91,108]; (**B**) synthesis of 2-aminophenoxazin-3-one derivatives [92] and (**C**) oxidation of sulphonamide derivatives of 3-hydroxyorthanilic acid and 3-amino-2-hydroxybenzenesulfonic acid by *T. versicolor* and *C. unicolor* laccases into symmetrically and non-symmetrically substituted phenoxazinones [94,95,96].

Phenoxazinone derivatives have been synthesized by fungal laccases of different origins [91,92,108]. In 1999, Osiadacz et al. reported the synthesis of cinnabarinic acid and 2-amino-4,6-dimethyl-3-phenoxazinone-1,9-carboxylic acid (actinocin), a pharmaceutical product proven to be effective in the fight against cancer, via a laccase-catalysed reaction from 3-hydroxyanthranlic acid (3-HAA) and 4-methyl-3-hydroxyanthranilic acid (4-M-3-HAA), respectively, as shown in Scheme 13A. The laccase isolated from *T. versicolor* was immobilized in polyacrylamide gel and the reaction performed at pH 5.0 in water and an acetonitrile/water mixture yielding actinocin with a 53% yield [91].

The synthesis of cinnabarinic acid and actinocin promoted by laccases was revisited by Giurg et al., who compared chemical and enzymatic oxidative methods for the oxidative homo-dimerizations of different 2-aminophenols promoted by laccases to afford the respective 2-aminophenoxazin-3-one derivatives (Scheme 13B). For the enzymatic methods, the best results were achieved with the air/laccase system which allow to obtain the correspondent 2-aminophenoxazin-3-ones in moderate to high yields (24–72%) [92].

Since then, other reports focused on the enzymatic condensation of *o*-aminophenols have been reported. The oxidative dimerization of 3-HAA and its sulfonated analog 3-hydroxyorthanilic acid (3-HOA), mediated by the fungal laccase from *P. cinnabarinus*, afforded cinnabarinic acid and the 2-amino-3-oxo-3H-phenoxazin-1,9-disulfonic acid, respectively [93]. Looking forward to the synthesis of a new class of water-soluble chromophores and potential bioactive molecules through a biocatalytic process, the oxidative homo- and cross-coupling reactions of numerous sulphonamide derivatives of 3-hydroxyorthanilic acid, as well as 3-amino-2-hydroxybenzenesulfonic acid, have been explored using the commercial laccase from *T. versicolor* leading to symmetrically and non-symmetrically substituted phenoxazinones [94,95]. The 3-amino-4-hydroxybenzene sulfonic acid has also been transformed to corresponding phenoxazinones via laccase-catalysed oxidative dimerization (Scheme 13C) [96].

**Scheme 14:**
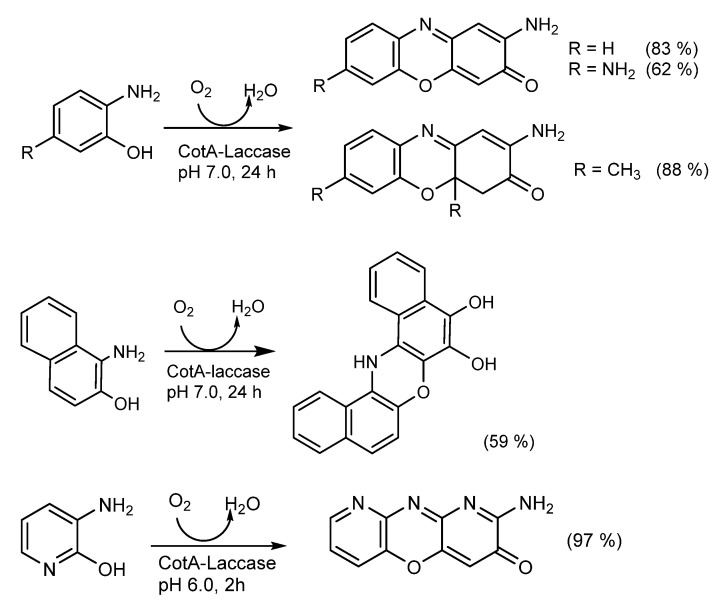
Synthesis of phenoxazinone-based products by oxidative homocoupling reactions of *o*-aminophenols mediated by CotA laccase [65,67].

More recently, we used the bacterial CotA-laccase to promote the biotransformation of diverse 2-aminophenol derivatives (2-aminophenol, 2,5-diaminophenol and 1-amino-2-naphthol) as model substrates. The corresponding phenoxazinone dyes were obtained within 2 h, in good to excellent yields (59–97%) (Scheme 14). The scope of the substrates oxidized by the CotA-laccase was further extended to a pyridine derivative yielding the correspondent pyridyloxazinone [65,67].

**Scheme 15:**
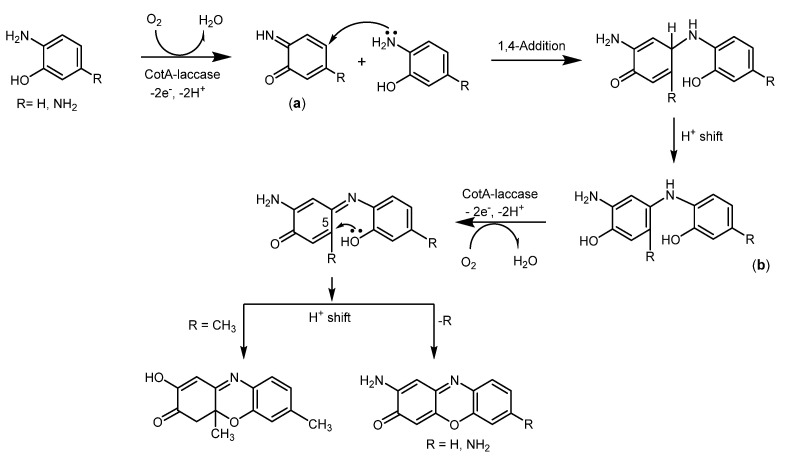
Proposed pathways involved in the formation of phenoxazinones from substituted aminophenols by CotA-laccase (adapted from [65,67]).

The mechanistic pathway of phenoxazinones biotransformation was revisited considering the redox properties of the substrates and their relative enzymatic rates of conversion (Scheme 15). The initial enzymatic step is the two successive one-electron oxidation of the *ortho*-aminophenols, generating *ortho*-quinone-imine intermediates (a). These oxidized electrophilic species suffer nucleophilic addition by another substrate molecule (or another similar substrate) followed by a proton shift, yielding the first coupling intermediate (b). A compound, non-substrate of the enzyme, could still act as a nucleophile to another susceptible *o*-aminophenol yielding a cross-coupled substituted phenoxazinone. The second two step one-electron enzymatic oxidation and an intramolecular Michael addition of the phenol group to the C5 atom, with the displacement of an R group, leads to a fully reduced aminophenoxazine, which is spontaneously oxidised in air to produce the final heterocycle product [65,94].

#### 3.2.4. Synthesis of Phenothiazine Derivatives

**Scheme 16:**
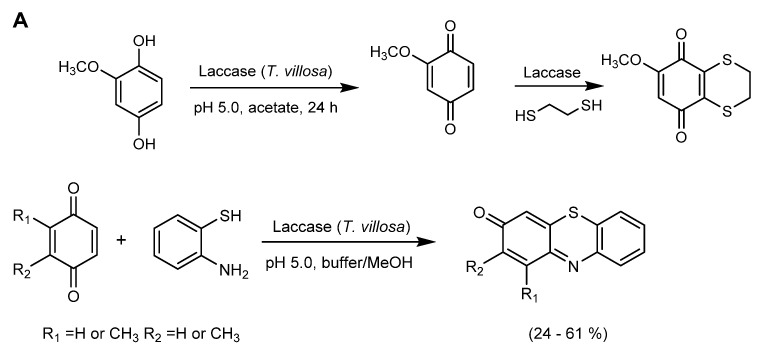

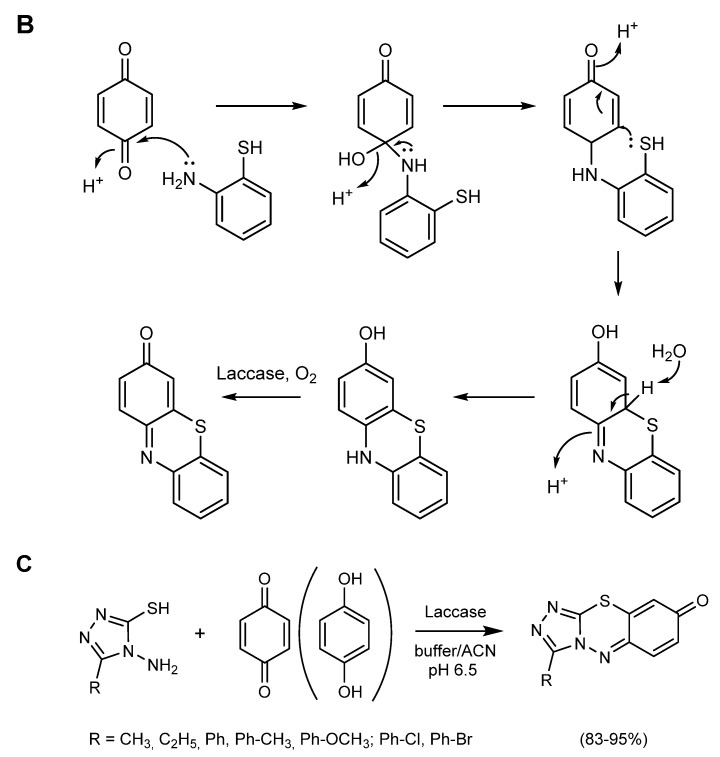
(**A**) Laccase-catalysed coupling reactions of 1,2-ethanedithiol or 2-aminothiophenol with 1,4-quinones; (**B**) Proposed reaction mechanism for the laccase-facilitated synthesis of phenothiazine derivatives [97]; (**C**) Laccase-catalysed reactions between quinones and substituted 4-amino-4H-1,2,4-triazolo-3-thiols [98].

Phenothiazines are heterocyclic sulphur compounds applicable in many areas of medicine, in particular in the treatment of neurodegenerative diseases such as Alzheimer’s and Parkinson’s diseases [74]. Considering that laccases can oxidize hydroquinones and catechols to produce in situ *p*- and *o*-quinones, the cross-coupling reactions involving sulphur-based nucleophiles (1,2-ethanedithiol or 2-aminothiophenol) were exploited providing a sustainable approach for the synthesis of 2,3-ethylenedithio1,4-quinone and phenothiazine substructures (Scheme 16A) [97,109].

The coupling between 1,4-quinones and 2-aminothiophenol mediated by the *T. villosa* laccase, yielded the correspondent phenothiazine derivatives at 24–61% yields (Scheme 16A) providing a sustainable approach for the synthesis of this biologically important class of compounds. However, relatively low yields were obtained (9–53%) when the reaction started from the hydroquinones, due to the competitive reaction of dimerization of 2-aminothiophenol [97].

Scheme 16B shows the reaction mechanism proposed, where the initial addition of the aromatic amino group to a carbonyl group of the 1,4-quinone yields the correspondent imine, followed by the addition of sulphur to an adjacent alkene carbon and subsequent tautomerization to produce the N,S-cyclic intermediate, which final oxidation results in the formation of the phenothiazine derivative.

Similar compounds were previously obtained by Bhalerao et al. through the reaction of benzoquinone, generated from hydroquinone by an oxidative in situ reaction with laccase, with various 5-substituted-4-amino-3-mercapto-1,2,4-triazoles (Scheme 16C). A mild and efficient one step synthesis of 3-substituted-1,2,4-triazolo(4,3-b)(4,1,2) benzothiadiazine-8-ones was proposed, giving rise to quantitative yields of corresponding products. In general, the yields of the products have been good and seem not to depend on the substitution pattern of the substrates [98].

## 4. Final Remarks

The use of laccases in organic synthesis is a promising alternative to the classical chemical oxidation methods resulting in the synthesis of a wide range of heterocyclic compounds. The variety of different aromatic scaffolds obtained by this enzymatic approach clearly shows that laccases are promising tools for both phenol and aromatic amines oxidation, boosting new eco-friendly alternatives to the production of value-added aromatic compounds. The inclusion of small quantities of green co-solvents is also well tolerated by the laccases, for the cases where the water solubility of the monomers is very low. This tolerance also allows the combination/integration of chemical and biocatalytic steps in the same synthetic route, broadening the scope of applications of laccases in organic synthesis. From an environmental point of view, the use of enzymes as biocatalysts is also critical, since the reactions can be carried out at ambient temperature in an aqueous medium, in accordance with principles of green chemistry. The increasing number of characterized laccases from different origin and displaying distinct properties, e.g., in the optimal pH and temperature, is also very auspicious for biotransformations relying on the activity of these enzymes. Moreover, the generation of tailor-made enzymes using protein engineering techniques also represents a proficient way to design highly efficient and stable biocatalysts required for handling other limiting factors such as thermostability, resistance to organic solvents, extremes of pH (acid or basic) and inhibitors.

## Figures and Tables

**Table 1:** Synthetic pathways for nitrogen- and oxygen-based heterocyclic compounds mediated by laccases at optimized reaction conditions.

Product	Enzyme	Laccase Microbial Source	Commercial	Reaction Conditions	Ref.
Five-membered rings					
Benzofuran derivatives	TvL	*Trametes versicolor*	--^1^	Acetate buffer (0.2 M), pH 4.37, r.t., 3–7 h, (51–99%)	[78]
	AbL	*Agaricus bisporus*	--^1^	Phosphate buffer (0.2 M), pH 6.0, r.t., 20–49 h, (88–99%)	[78]
	TvL	*Trametes versicolor*	Fluka, Buchs	Acetate buffer, pH 4.38, r.t., 5h, 67%	[79]
	AbL	*Agaricus bisporus*	Fluka, Buchs	Phosphate buffer (0.2 M), pH 5.96, r.t., 18–24 h, (70–97%)	[79]
	AbL	*Agaricus bisporus*	--^1^	Phosphate buffer (0.2 M), pH 6.0, r.t., 17–24 h, (55–98%)	[80]
	MtL	*Myceliophthora thermophila*	Suberase^®^, Novozymes	Phosphate buffer (0.1 M), pH 7.15, r.t., 24 h, (37–98%)	[81,82]
	PcL	*Pycnoporus cinnabarinus*	--^2^	O_2_, phosphate/citrate buffers (0.1 M), pH 7.0, HBT, r.t., 12 h, (39–65%)	[83]
2-arylbenzimidazoles	--	--	Novoprime Base 268, Novozymes	Acetate buffer (0.1 M): CH_3_CN (50:50), pH 4.0, r.t., 2–24 h, (56–88%)	[10]
	AbL	*Agaricus bisporus*	Fluka	Phosphate buffer (0.2 M) or buffer: methanol (5:2), pH 6.0, r.t., 3–18 h, (50–99%)	[72]
	TvL	*Trametes versicolor*	Sigma-Aldrich	TvL: TEMPO immobilized on magnetic iron (II,III) oxide nanoparticles heterogeneous catalyst, citrate buffer (10 mM), pH 4.5, 40 °C, 10 h, (69–88%)	[84]
Benzothiazole derivatives	--	--	Novoprime Base 268, Novozymes	Acetate buffer (0.1 M): CH_3_CN (50:50), pH 4.0, 25 °C, 24 h, (48–88%)	[10]
	TvL	*Trametes versicolor*	Sigma-Aldrich	TvL: DDQ (10% mol) catalytic system, phosphate buffer (0.1 M):CH_3_CN (4%), pH 5.0, 45 °C, 24h, (65–98%)	[85]
	AbL	*Agaricus bisporus*	ASA Spezialenzyme	Phosphate buffer (0.2 M): ethanol (10%), pH 6.0, r.t., 12–28 h, (78–97%)	[86]
Six-membered rings					
Quinazoline and quinazolinone derivatives	TvL	*Trametes versicolor*	Sigma-Aldrich	O_2_ or air, TvL: DDQ catalytic system, phosphate buffer (0.1 M): CH_3_CN (4%), pH 4.5, 45 °C, 24 h, (80–95%)	[85]
	TvL	*Trametes versicolor*	Sigma-Aldrich	O_2_ or air, TvL: DBTC or TvL/TEMPO catalytic systems, phosphate buffer (0.1 M): CH_3_CN (4%), pH 4.5, 45 °C, 20–24 h, (40–96%)	[87]
Phenazines	MtL	*Miceliophthora termophila*	--^1^	Britton–Robinson buffer (0.1 M), pH 5.0, 60 °C, 1h	[88]
	CotA- laccase	*Bacillus subtilis*	--^2^	Phosphate buffer (0.1 M): ethanol (10%), pH 6–7, r.t., 2–24 h, (30–96%)	[65,67]
	PoL	*Pleurotus ostreatus*	--^2^	Free PoL and immobilised on porous Purolite^®^ carriers, tartrate buffer (40 mM), pH 5.5, 28 °C, 48h	[89]
	LAC	*Cerrena unicolor*	--^2^	Air, tartrate buffer (0.1 M), pH 4.0–4.5, 28 °C, 72 h, (19–27%)	[90]
Phenoxazines and phenoxazinones	TvL	*Trametes versicolor*	--^2^	Free and immobilized TvL on polyacrylamide gel, phosphate buffer (0.1 M), pH 5.0, 25 °C, 1 h, (38–74%)	[91]
	TvL	*Trametes versicolor*	--^2^	Phosphate buffer (0.066 M): methanol (2%), pH 5.0, 20 °C, 0.5–23 h, (24–72%)	[92]
	TvL	*Trametes versicolor*	Oxyzym LA, Bioscreen e.K	Phosphate buffer (0.1 M), pH 6–7, 25 °C, 24 h, (75–90%)	[93]
	TvL	*Trametes versicolor*	Sigma-Aldrich Oxyzym LA, Bioscreen e.K	Acetate buffer (0.2 M): methanol (5%), pH 6.0, 25 °C, 16 h	[94]
	TvL	*Trametes versicolor*	Oxyzym LA, Bioscreen e.K	Acetate buffer (0.2 M): methanol (5%), pH 4–6, 25 °C, 24h, (40–93%)	[95]
	CuL	*Cerrena unicolor*	--^2^	Tartrate buffer (0.1 M), pH 5, 25 °C, 24 h	[96]
	CotA- laccase	*Bacillus subtilis*	--^2^	Phosphate buffer (0.1M): ethanol (10%), pH 6–7, r.t., 2–24 h, (59–97%)	[65,67]
Phenothiazine derivatives	TvL	*Trametes villosa*	Novo Nordisk Biochem	Acetate buffer (0.1 M): methanol (15%), pH 5.0, r.t., 6 h, (24–61%)	[97]
		Not mentioned	Sigma-Aldrich	Phosphate buffer: CH_3_CN (3:1), pH 6.5, 12 h, (83–95%)	[98]

^1^ Not mentioned; ^2^ Not commercial. DDQ—2,3-dichloro-5,6-dicyano-1,4-benzoquinone; DBTC—3,5-di-tert-butylcathecol.
